# Clinical characteristics and risk factors of hospital mortality in elderly patients with community-acquired pneumonia

**DOI:** 10.3389/fmed.2025.1512288

**Published:** 2025-03-19

**Authors:** Shasha Li, Lu Li, Shengyu Wang, Hao Wu

**Affiliations:** ^1^Department of Nephrology, First Affiliated Hospital of Xi’an Medical University, Xi’an, Shaanxi, China; ^2^Department of Respiratory, First Affiliated Hospital of Xi’an Medical University, Xi’an, Shaanxi, China

**Keywords:** clinical characteristics, risk factors, hospital mortality, elderly patients, CAP

## Abstract

**Background:**

Community-acquired pneumonia (CAP) leads to high morbidity and mortality among the elderly, with 3 million deaths annually worldwide. Multiple comorbidities significantly increase the risk. This study aims to identify independent risk factors for mortality in elderly patients with CAP to optimize individualized treatment strategies.

**Methods:**

This single-center retrospective study was conducted at First Affiliated Hospital of Xi’an Medical University. Clinical data from elderly patients diagnosed with CAP between December 2018 and December 2023 were retrospectively collected. Logistic regression analysis was used to determine risk factors for in-hospital mortality. A nomogram was constructed based on the final model for risk assessment.

**Results:**

A total of 613 eligible patients were included, with 68.2% being male, and a median age of 78 (IQR 70–86) years. The prevalence of hypertension, coronary heart disease (CHD), stroke, diabetes, malignancy, and chronic obstructive pulmonary disease (COPD) was 55.5, 39.8, 29.5, 27, 16.6, and 7%, respectively. The in-hospital mortality rate was 48%. Compared to survivors, non-survivors were older, had a higher proportion of males, faster heart rates, and higher rates of comorbidities. Multivariate logistic regression analysis identified age (OR 1.05, 95% CI [1.02–1.07], *P* < 0.01), BMI (OR 0.92, 95% CI [0.86–0.98], *P* < 0.01), stroke (OR 2.21, 95% [1.43–3.42], *P* < 0.01), ARDS (OR 4.0, 95% CI [2.17–7.37], *P* < 0.01), AKI (OR 2.98, 95% CI [1.77–5.01], *P* < 0.01), malignancy (OR 2.11, 95% CI [1.22–3.65], *P* < 0.01), elevated WBC (OR 1.20, 95% [1.14–1.27], *P* < 0.01), PLT (OR 0.995, 95% CI [0.993–0.998], *P* < 0.01), and albumin (OR 0.93, 95% CI [0.90–0.97], *P* < 0.01) as independent risk factors for in-hospital mortality. The area under the curve (AUC) of the multivariable model was 0.85 (95% CI [0.81–0.87], *P* < 0.01).

**Conclusion:**

Elderly CAP patients have a high prevalence of comorbidities and a high in-hospital mortality rate. Advanced age, low BMI, stroke, ARDS, AKI, malignancy, elevated WBC, decreased PLT, and low albumin were independent risk factors for in-hospital mortality.

## 1 Introduction

Community-acquired pneumonia (CAP) is one of the leading causes of morbidity and mortality among elderly populations worldwide (1). More than 1 million deaths from pneumonia occur each year globally, with an overall hospital mortality rate associated with CAP ranging between 20% and 50%. Mortality is particularly higher among those aged 65 years and older (2–4). A multicenter study in the US found that the estimated hospitalization rate for CAP in adults aged 50–64, 65–79, and 80 years or older was approximately 4, 9, and 25 times higher than that in adults aged 18–49 years (2). European studies have shown that CAP incidence rates in hospitalized and outpatient settings range from 50 to 2,940 per 100,000 people and 45–2,380 per 100,000 people, respectively, with a mortality rate of 70 per 100,000 (5). As the global population ages, the health burden of CAP on the elderly continues to increase, making CAP management a critical clinical challenge.

The incidence and hospitalization rates for elderly CAP patients are on the rise, yet studies on the epidemiological characteristics and mortality risk factors in this population remain limited. Previous research has shown that elderly CAP patients often have multiple chronic conditions, such as hypertension, coronary heart disease, diabetes, chronic obstructive pulmonary disease (COPD), stroke, and malignancy. These comorbidities not only complicate the clinical course but also significantly increase mortality risk (6). Despite this, studies on in-hospital mortality among elderly CAP patients have limitations in sample size, data analysis methods, and application of clinical prediction models.

Existing research has explored some mortality risk factors in elderly CAP patients. For example, Zhong et al. (7) identified chest tightness, prolonged fever, low serum albumin, and low sodium as factors associated with CAP deterioration in a retrospective study. Glöckner et al. (8) highlighted advanced age, low BMI, diabetes, chronic kidney disease, and chronic neurological diseases as predictors of early post-discharge mortality in CAP patients. However, many of these studies focus on single risk factors, lacking systematic multivariate analyses and individualized risk assessment tools, which limit their clinical applicability.

Therefore, we conducted a retrospective observational study to systematically analyze the clinical characteristics of elderly CAP patients and identify independent risk factors for in-hospital mortality. By identifying these risk factors, we aim to provide a basis for early clinical intervention and optimize individualized treatment strategies for elderly CAP patients.

## 2 Materials and methods

### 2.1 Study design

This retrospective observational study was conducted at the First Affiliated Hospital of Xi’an Medical University. The study adhered to the ethical principles of the Helsinki Declaration (2013) (9). Informed consent was waived due to the retrospective and anonymous nature of the data. The study was reported according to the STROBE guidelines for observational studies (10).

### 2.2 Participants

We retrospectively screened all patients discharged from the First Affiliated Hospital of Xi’an Medical University with a diagnosis of CAP between December 2018 and December 2023. Inclusion criteria were: (1) Age 60 years and older; (2) Diagnosed with CAP during hospitalization, confirmed by radiographic findings (e.g., chest X-ray or CT) or consistent clinical symptoms (e.g., cough, fever, dyspnea). Exclusion criteria included: (1) Pneumonia due to other causes (e.g., hospital-acquired pneumonia, fungal infections, tuberculosis); (2) Severe organ failure upon admission; (3) Incomplete assessments during hospitalization; (4) Lack of follow-up data.

### 2.3 Data collection

Potential risk factors for in-hospital mortality were collected from electronic medical records, including demographic information, comorbidities, vital signs, CAP-related complications, and laboratory results.

### 2.4 Definition

The main complications of community-acquired pneumonia include acute respiratory distress syndrome (ARDS) and acute renal injury (AKI). The diagnostic criteria of ARDS rely on the 2012 Berlin definition (11): (1) Mild: 200 mm Hg < PaO_2_/FIO_2_ ≤ 300 mm Hg with PEEP or CPAP ≥ 5 cmH2O; (2) Moderate: 100 mmHg < PaO_2_/FIO_2_ ≤ 200 mmHg with PEEP ≥ 5 cmH2O; and Severe: PaO_2_/FIO_2_ ≤ 100 mmHg with PEEP ≥ 5 cmH2O. AKI was diagnosed according to the 2012 Kidney Disease: Improving Global Outcomes (KDIGO) guideline (12): Increase in sCr by ≥ 50% within 7 days or increase in sCr by ≥ 0.3 mg/dl (26.5 μmol/l) within 2 days or oliguria for ≥ 6 h.

### 2.5 Research results

The results include mortality, clinical features and risk factors of in-hospital mortality.

### 2.6 Statistical analysis

Depending on the normality of distribution, continuous variables were expressed as mean ± standard deviation (SD) or median (interquartile interval [IQR]) and were compared by using the Student’s *t*-test and Mann-Whitney U test, respectively. Categorical variables were presented as count (percentage) and were compared using chi square test or Fisher exact probability test where appropriate.

The risk factors for in-hospital mortality were identified by using logistic regression analysis. Initially, univariate logistic regression analyses were performed for each of the candidate predictors. The factors that were significantly associated with in-hospital mortality in the univariate logistic regression analyses (*P* < 0.05) were included in the multivariate logistic regression model. The variables that were highly correlated or associated with each other were excluded from the model by using multicollinearity analysis (VIF > 10). Thereafter, the stepwise regression method was employed for variable screening to establish the final model. The discrimination performance of the model was assessed by using the receiver operating characteristic curve (ROC) analysis and area under ROC curve (AUC). To facilitate risk assessment, a nomogram based on the independent risk factors remaining in the final model was constructed. The level of significance was set at 0.05 (two-tailed). All the statistical analyses were performed by using the R software (13).

## 3 Results

### 3.1 Clinical characteristics of the included patients

**FIGURE 1:**
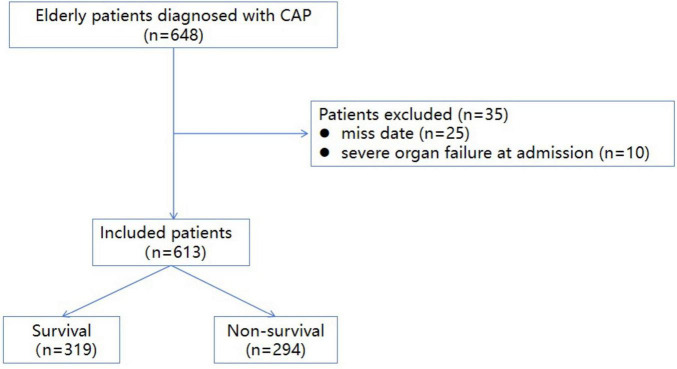
The inclusion flow chart of included patients (A flowchart illustrating the process of patient selection and inclusion in the study. The diagram details the number of patients screened, excluded, and ultimately included in the analysis, along with the reasons for exclusion at each stage).

The baseline characteristics of the included patients were detailed in Table 1. During the study period, a total of 648 elderly patients were admitted for CAP in our hospital. After screening, 35 patients were excluded and 613 eligible patients were finally included (Figure 1). The study cohort comprised 613 patients with community-acquired pneumonia, stratified into three age groups: 60–70 years (*n* = 174, 28.4%), 70–80 years (*n* = 243, 39.6%), and > 80 years (*n* = 196, 32.0%). Mortality rates demonstrated a significant age-dependent escalation, with 35.6% (62/174) in the 60–70 years group, 42.4% (103/243) in the 70–80 years group, and 65.3% (128/196) in the > 80 years group. The male proportion of the included patients was 62.8% (385/613) with a median age of 78 (IQR 70–86) years. The prevalence of hypertension, coronary heart disease (CHD), stroke, diabetes, tumor, and chronic obstructive pulmonary disease (COPD) among the included patients was 55.5, 39.8, 29.5, 27, 16.6, and 7%, respectively. The in-hospital mortality rate of included patients was 48% (294/613). The most common cause of death was severe pneumonia (33.6%) followed by heart failure (15.6%), respiratory and circulatory failure (12.5%), stroke (8.1%), respiratory failure (7.4%), and septic shock (7.1%) (Table 2).

**TABLE 1:** Baseline characteristics of the included patients.

Characteristics	Overall (*N* = 613)	Survival (*n* = 319)	Non-survival (*n* = 294)	*P*
Male	385 (62.8)	187 (58.6)	198 (67.3)	0.02
Age (year)	78 (70–86)	75 (67–83)	83 (74–87)	< 0.01
BMI (kg/m^2^)	22 (20–24)	22.5 (20.4–24.4)	21.4 (19–23.4)	< 0.01
MAP (mmHg)	93 (83–100)	93 (83–101)	91 (80–98)	0.01
Heart rate (bpm)	80 (76–90)	80 (75–88)	84 (78–100)	< 0.01
Smoke	92 (15)	53 (16.6)	39 (13.2)	0.24
**Comorbidities**				
Diabetes	165 (27)	74 (23.2)	91 (31)	0.03
Hypertension	340 (55.5)	168 (52.6)	172 (58.5)	0.14
Stroke	181 (29.5)	66 (20.6)	115 (39.1)	< 0.01
CHD	244 (39.8)	132 (41.3)	112 (38.1)	0.40
ARDS	81 (13.2)	20 (6.2)	61 (20.7)	< 0.01
AKI	137 (22.3)	31 (9.7)	106 (36)	< 0.01
Tumor	102 (16.6)	41 (12.8)	61 (20.7)	< 0.01
COPD	43 (7)	23 (7.2)	20 (6.8)	0.84
**Laboratory tests**				
WBC (10^9^/L)	7.03 (4.76–10.28)	5.74 (4.38–7.85)	8.94 (5.73–13.1)	< 0.01
Hb (g/L)	117 (100–132)	120 (106–132)	114 (96–130)	< 0.01
PLT (10^9^/L)	174 (125–225)	184 (133–237)	165 (113–213)	< 0.01
TBil (μmol/L)	14.2 (10.2–18.7)	13.4 (10.1–18)	15 (10.2–21.2)	< 0.01
Albumin (g/L)	32.3 (28.6–36.1)	34 (31.2–37.5)	30.8 (27.2–33.7)	< 0.01
Scr (μmol/L)	85 (61–143)	77 (58–143)	100 (68–159)	< 0.01
D-dimer (mg/L)	1.83 (0.71–5.6)	1.03 (0.47–5.6)	3.11 (1.29–5.6)	< 0.01

Data were expressed as *n* (%) or median (IQR). AKI, acute kidney injury; ARDS, acute respiratory distress syndrome; BMI, body mass index; COPD, chronic obstructive pulmonary disease; CHD, coronary heart disease; Hb, hemoglobin; IQR, interquartile range; PLT, platelet; MAP, mean arterial pressure; WBC, white blood cell.

**TABLE 2:** Causes of death in the study population.

Causes	Proportion
Severe Pneumonia	99 (33.6)
Heart failure	46 (15.6)
Respiratory and circulatory failure	37 (12.5)
Stroke	24 (8.1)
Respiratory failure	22 (7.4)
Septic shock	21 (7.1)
Cancer	11 (3.7)
Acute myocardial infarction	8 (2.7)
Renal failure	4 (1.3)
Cardiogenic shock	4 (1.3)
Cardiac arrest	4 (1.3)
Liver failure	3 (1)
Gastrointestinal bleeding	3 (1)

Data were expressed as *n* (%).

### 3.2 Risk factors for in-hospital mortality

#### 3.2.1 Univariate analysis results

In non-survivors, the age (83 [74–87] years vs. 75 [67–83] years, *P* < 0.01), male proportion (67.3% vs. 58.6%, *P* = 0.02), and heart rate (HR) (84 [78–100] bpm vs. 80 [75–88] bpm, *P* < 0.01) were significantly higher compared with the survivors, whereas survivors had greater BMI (22.5 [20.4–24.4] kg/m^2^ vs. 21.4 [19–23.4] kg/m^2^, *P* < 0.01) and mean arterial pressure (MAP) (93 [83–101] mmHg vs. 91 [80–98] mmHg, *P* = 0.01). The non-survivors had higher prevalence in terms of diabetes (31% vs. 23.2%, *P* = 0.03), stroke (39.1% vs. 20.6%, *P* < 0.01) and tumor (20.7% vs. 12.8%, *P* < 0.01) compared with the survivors. There was no significant difference in the prevalence of smoking, hypertension, CHD, and COPD between the two groups. More patients in the non-survivors group developed CAP associated complications including ARDS (20.7% vs. 6.2%, *P* < 0.01) and AKI (36% vs. 9.7%, *P* < 0.01) (Table 1).

#### 3.2.2 Multivariate analysis results

**FIGURE 2:**
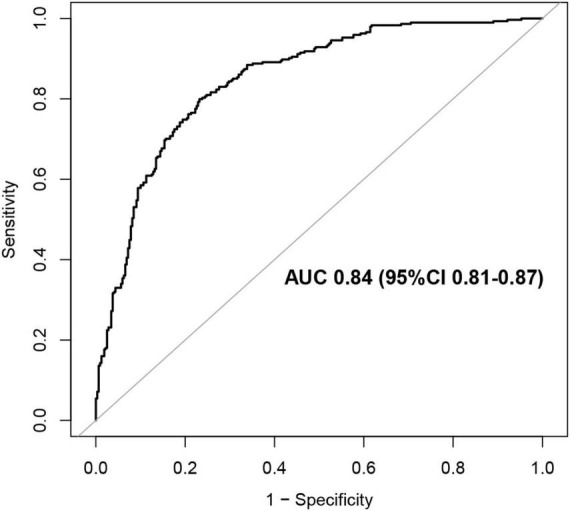
Receiver operating characteristic (ROC) curve of the final logistic regression model [The ROC curve demonstrates the diagnostic performance of the final logistic regression model. The area under the curve (AUC) is provided as a measure of the model’s predictive accuracy].

**FIGURE 3:**
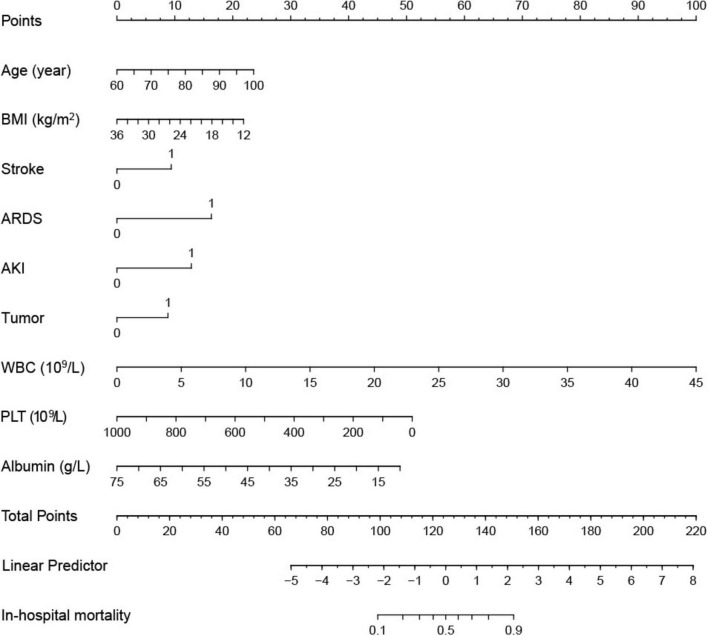
A nomogram constructed from the final logistic regression model, enabling the visualization and calculation of individual patient risk scores based on the model’s predictors (Each predictor is assigned a point value, and the total points correspond to the predicted probability of the outcome).

Multivariate logistic regression analysis identified advanced age (OR 1.05, 95% CI [1.02–1.07], *P* < 0.01), lower BMI (OR 0.92, 95% CI [0.86–0.98], *P* < 0.01), stroke (OR 2.21, 95% [1.43–3.42], *P* < 0.01), ARDS (OR 4.0, 95% CI [2.17–7.37], *P* < 0.01), AKI (OR 2.98, 95% CI [1.77–5.01], *P* < 0.01), Tumor (OR 2.11, 95% [1.22–3.65], *P* < 0.01), elevated WBC (OR 1.20, 95% [1.14–1.27], *P* < 0.01), and decreased PLT (OR 0.995, 95% CI [0.993–0.998], *P* < 0.01) or Albumin (OR 0.93, 95% CI [0.90–0.97], *P* < 0.01) as risk factors independently associated with in-hospital mortality (Table 3). The area under the curve (AUC) of the multivariable model was 0.85 (95% CI [0.81–0.87], *P* < 0.01) (Figure 2). The nomogram based on the final model was showed in Figure 3.

**TABLE 3:** The results of univariate and multivariate logistic regression analysis.

Variables	Univariate analysis				Multivariate analysis			
	**Coefficient**	**OR**	**95% CI**	***P***	**Coefficient**	**OR**	**95% CI**	***P***
Age (year)	0.066841	1.06	1.04–1.08	< 0.01	0.050238	1.05	1.02–1.07	< 0.01
Male gender	0.37561	1.45	1.04–2.02	0.02				
BMI (kg/m^2^)	−0.11132	0.89	0.84–0.94	< 0.01	−0.077554	0.92	0.86–0.98	0.01
MAP (mmHg)	−0.011011	0.98	0.97–0.99	0.03				
HR (bpm)	0.023805	1.02	1.01–1.03	< 0.01				
Smoke	−0.26450	0.76	0.49–1.20	0.24				
DM	0.39485	1.48	1.03–2.12	0.03				
Hypertension	0.23679	1.26	0.92–1.74	0.14				
CHD	−0.13720	0.87	0.63–1.20	0.40				
Stroke	0.90128	2.46	1.72–3.52	< 0.01	0.79702	2.21	1.43–3.42	< 0.01
ARDS	1.36455	3.91	2.29–6.67	< 0.01	1.38712	4.00	2.17–7.37	< 0.01
AKI	1.65597	5.23	3.37–8.13	< 0.01	1.09275	2.98	1.77–5.01	< 0.01
Tumor	0.57388	1.77	1.15–2.73	< 0.01	0.75025	2.11	1.22–3.65	< 0.01
COPD	−0.062531	0.93	0.50–1.74	0.84				
WBC (10^9^/L)	0.18146	1.19	1.14–1.25	< 0.01	0.18918	1.20	1.14–1.27	< 0.01
Hb (g/L)	−0.010875	0.98	0.98–0.99	< 0.01				
PLT (10^9^/L)	−0.0029059	0.99	0.99–0.99	< 0.01	−0.0043407	0.995	0.993–0.998	< 0.01
TBil (μmol/L)	0.0056481	1.005	0.99–1.01	0.16				
Albumin (g/L)	−0.12028	0.88	0.85–0.91	< 0.01	−0.064018	0.93	0.90–0.97	< 0.01
Scr (μmol/L)	0.00085629	1.0009	0.99 to 1.001	0.07				
D-dimer (mg/L)	0.060232	1.06	1.03–1.09	< 0.01				

AKI, acute kidney injury; ARDS, Acute Respiratory Distress Syndrome; BMI, body mass index; CHD, coronary heart disease; COPD, Chronic Obstructive Pulmonary Disease; DM, diabetes mellitus; Hb, hemoglobin; HR, heart rate; MAP, mean arterial pressure; PLT, platelet; TBil, total bilirubin; WBC, white blood cell.

## 4 Discussion

In this retrospective observational study of 613 elderly patients hospitalized with CAP, we found an in-hospital mortality rate as high as 48%. This finding aligns with the growing recognition that older adults are particularly vulnerable to severe respiratory infections, especially in the presence of multiple comorbidities. Our multivariate analysis identified advanced age, lower BMI, stroke, ARDS, AKI, malignancy, WBC count, and PLT or albumin levels as independent risk factors associated with mortality. Based on these variables, we developed a predictive nomogram that provides a clinically useful tool for risk stratification.

The overall mortality observed in our study is consistent with earlier research on CAP, reflecting the heightened risk faced by the elderly population. Pieralli et al. (14) highlighted a 30-day mortality rate exceeding 15% in elderly CAP patients, emphasizing the dual challenge posed by advanced age and comorbidities. Our findings reinforce the critical nature of advanced age as a predictor of poor prognosis, with mortality risk increasing by 5% for each additional year of age. This observation is corroborated by a study by Zan et al. (15), which included 304 CAP patients aged 90 and above, demonstrating a significantly elevated mortality risk in older age groups, particularly those over 90 years old.

Furthermore, our study contributes to a deeper understanding of the relationship between BMI and mortality in this population. While previous research documented no significant difference in in-hospital or out-of-hospital mortality when BMI was < 18.5 kg/m^2^ or ≥ 24 kg/m^2^ (16), our results show that lower BMI is significantly associated with increased mortality in the context of CAP. This is consistent with evidence suggesting that underweight individuals face a higher risk of adverse outcomes due to frailty and reduced physiological reserves (17). The majority of patients in our cohort had a BMI within the normal range, which may explain this protective association, indicating that strategies to address malnutrition in elderly patients may be key.

Comorbidities such as stroke and malignancy emerged as important factors influencing mortality risk. The interplay between CAP pathophysiology and the identified risk factors warrants specific elaboration. First, the inclusion of both historical and incident tumors in our analysis reflects the dual mechanisms by which malignancy impacts pneumonia outcomes: (1) chronic immune dysregulation from hematologic malignancies or chemotherapy-induced lymphopenia (18), and (2) acute metabolic stress from paraneoplastic syndromes exacerbating hypoxemia. Regarding stroke temporalities, pre-existing cerebrovascular disease may predispose to aspiration pneumonia through impaired cough reflex (19), while in-hospital strokes likely represent thromboinflammatory complications of CAP itself (line 213–222). Our findings indicate that stroke increases the risk of in-hospital mortality by 2.21 times, which echoes results from meta-analyses showing cerebrovascular disease as a significant predictor of poor prognosis in pneumonia patients (11, 12). Similarly, the presence of malignancy raised the mortality risk by 1.11 times, highlighting the need for personalized management strategies that account for the complex interplay of these comorbidities.

The high incidence of ARDS and AKI observed in our cohort further underscores the need for vigilance in monitoring these complications. The incidence rates of ARDS and AKI in our study were 13.2% and 22.3%, respectively, but in deceased patients, the rates were as high as 20.7% and 36%. These rates are lower than those reported by Xie et al. (12), who found ARDS and AKI rates of 38% and 39.7%, which may be due to our focus on elderly patients. As in our study, ARDS and AKI are recognized as significant contributors to mortality in pneumonia patients. The pooled mortality estimates for patients with ARDS and AKI emphasize the need for timely and aggressive interventions, such as mechanical ventilation and intensive care support.

Our findings carry significant clinical implications. The AUC for the nomogram we developed was 0.85, providing a robust framework for early identification of patients at high risk of mortality. By facilitating risk assessment, this tool can assist clinicians in making informed decisions about interventions and resource allocation, potentially reducing mortality in this vulnerable population. Additionally, proactive management of identified risk factors, such as providing nutritional support to patients with low BMI and closely monitoring for ARDS and AKI, could improve outcomes.

### 4.1 Limitations

The main limitation of this study is the low etiological diagnosis rate, which is related to the following factors: (1) According to the CAP diagnosis and treatment guidelines, mild patients usually only receive empirical treatment without compulsory pathogen detection (20); (2) Antibiotic pretreatment may reduce the positive rate of microbial culture (21). Nevertheless, this study focuses on the relationship between clinically accessible routine indicators (such as age, complications, and inflammatory markers) and prognosis. These indicators remain of practical value for rapid bedside risk assessment when the pathogen is unknown. Even in high-resource medical environments, 30%–50% of CAP cases cannot identify the pathogen (2). The results of this study suggest that in the absence of accurate microbiological data, strengthening early intervention for elderly patients and those complicated with respiratory failure may improve the prognosis.

Despite the strengths of this study, there are several limitations. The retrospective design and single-center nature may limit the generalizability of our findings. Although we employed strict inclusion criteria, the mortality rates might be influenced by the severity of cases seen at our institution. Moreover, the data collected were not comprehensive; important clinical features and laboratory tests were limited, which may have led to the omission of other relevant risk factors.

Additionally, while we conducted multivariate analyses to identify independent predictors, potential confounding variables may not have been fully accounted for. Lastly, though we assessed the predictive performance of the nomogram, external validation in different populations is crucial to confirm its applicability and reliability in clinical practice. Future research should also explore the underlying mechanisms driving the observed associations, particularly the role of nutritional status and its impact on clinical outcomes.

Our study underscores the significant interaction of age, BMI, and comorbidities in determining mortality in elderly patients with community-acquired pneumonia (CAP). These findings call for tailored management approaches for this population, emphasizing the integration of risk factors into clinical decision-making. By adopting these strategies, healthcare providers can enhance the care of elderly patients, ultimately aiming to improve outcomes and reduce the mortality associated with this prevalent disease.

## 5 Conclusion

Elderly patients with CAP have a high prevalence of comorbidities and an elevated in-hospital mortality rate. Advanced age, low BMI, stroke, ARDS, AKI, malignancy, elevated white blood cell count, and reduced platelet count or albumin levels are independent risk factors for in-hospital mortality in this population. Further studies with external validation and longitudinal follow-up are needed to confirm our findings.

## Data Availability

The raw data supporting the conclusions of this article will be made available by the authors, without undue reservation.
